# Cholesterol efflux alterations in adolescent obesity: role of adipose-derived extracellular vesical microRNAs

**DOI:** 10.1186/s12967-019-1980-6

**Published:** 2019-07-22

**Authors:** Matthew D. Barberio, Lora J. Kasselman, Martin P. Playford, Samuel B. Epstein, Heather A. Renna, Madeleine Goldberg, Joshua DeLeon, Iryna Voloshyna, Ashley Barlev, Michael Salama, Sarah C. Ferrante, Evan P. Nadler, Nehal Mehta, Allison B. Reiss, Robert J. Freishtat

**Affiliations:** 1 Center for Genetic Medicine Research, Children’s Research Institute, Children’s National Health System, Washington, DC 20010 USA; 2 Winthrop Research Institute and Department of Medicine, NYU Winthrop Hospital, 101 Mineola Boulevard, Suite 4-004, Mineola, NY 11501 USA; 3 National Heart Lung and Blood Institute, Bethesda, MD USA

**Keywords:** Extracellular vesicle, microRNAs, Obesity, Cholesterol Efflux

## Abstract

**Background:**

Macrophage cholesterol efflux capacity has been identified as a predictor for cardiovascular disease. We assessed the relationship between adipocyte-derived extracellular vesicle microRNAs and macrophage cholesterol efflux capacity.

**Methods:**

We assessed an adolescent cohort (n = 93, Age, median (IQR) = 17 (3) year, Female = 71, Male = 22) throughout the BMI continuum (BMI = 45.2 (13.2) kg/m^2^) for: (1) cholesterol efflux capacity and lipoprotein profiles; (2) adipocyte-derived extracellular vesicle microRNAs in serum; (3) the role of visceral adipose tissue extracellular vesicle in regulation of cholesterol efflux and cholesterol efflux gene expression in THP-1 macrophages in vitro.

**Results:**

Efflux capacity was significantly associated with HDL (r = 0.30, p = 0.01) and LDL (r = 0.33, p = 0.005) particle size. Multivariate-analysis identified six microRNAs associated (p < 0.05) with cholesterol efflux capacity: miR-3129-5p (Beta = 0.695), miR-20b (0.430), miR9-5p (0.111), miR-320d (− 0.190), miR301a-5p (0.042), miR-155-5p (0.004). In response to increasing concentrations (1 μg/mL vs. 3 μg/mL) of VAT extracellular vesicle, cholesterol efflux (66% ± 10% vs. 49% ± 2%; p < 0.01) and expression of ABCA1 (FC = 1.9 ± 0.8 vs 0.5 ± 0.2; p < 0.001), CD36 (0.7 ± 0.4 vs. 2.1 ± 0.8, p = 0.02), CYP27A1 (1.4 ± 0.4 vs. 0.9 ± 0.5; p < 0.05), and LXRA (1.8 ± 1.1 vs. 0.5 ± 0.2; p < 0.05) was altered in THP-1 cells in vitro.

**Conclusion:**

Adipocyte-derived extracellular vesicle microRNAs may, in part, be involved macrophage cholesterol efflux regulation.

**Electronic supplementary material:**

The online version of this article (10.1186/s12967-019-1980-6) contains supplementary material, which is available to authorized users.

## Background

Atherosclerotic cardiovascular disease (ASCVD) remains the leading cause of morbidity and mortality worldwide [1]. Although primarily a disease of adults, youth with obesity show evidence of subclinical ASCVD [1–3] which places them at increased risk as adults for coronary heart disease [2] and stroke [4]. The mechanisms by which obesity confers cardiovascular risk are not fully understood, but inflammation within visceral adipose tissue (VAT) is thought to be contributory [5, 6]. Further, the impact of excess adipose tissue on distal sites such as arterial wall monocytes/macrophages, direct participants in ASCVD, are also thought to contribute to disease pathogenesis [7].

Development of ASCVD is characterized by macrophage lipid overload leading to the formation of foam cells, and factors that accelerate this process are deemed atherogenic [8]. Macrophage cholesterol homeostasis is a delicate balance between influx, endogenous synthesis, esterification and hydrolysis, and efflux [9, 10]. Reduction in cholesterol efflux from macrophages is inversely related to carotid intima-media thickness, elevating the likelihood of the development of ASCVD [11]. Like other systemic inflammatory conditions psoriasis [12] and rheumatoid arthritis [10], obesity is a risk factor for ASCVD, but the mechanistic link between excess adiposity and ASCVD remains poorly understood [6].

In an effort to determine how adipose tissue affects distant cells and tissues, we identified adipocyte-derived exosomes as a potential link between obesity and its comorbidities [13–15]. Extracellular vesicles (EVs) are microvesicles that allow intercellular communication, carrying signaling molecules such as proteins and nucleic acids, including functional mRNA and microRNA [16]. We previously showed that adipocyte-derived EV microRNA content is pathologically altered by obesity and reversed by weight-loss surgery [13, 15]. A growing line of evidence from animal studies show that exosome-like vesicles released from adipose tissue carry the majority of circulating microRNAs [17] and are capable of pro-atherogenic effects [18].

Therefore, we sought determine the relationship between macrophage cholesterol efflux capacity and circulating adipocyte-derived EV microRNAs. We also sought to determine if exposure to VAT EVs regulated macrophage cholesterol efflux and cholesterol efflux gene expression in vitro. We hypothesized that exosomal microRNAs targeting established cholesterol efflux genes (ABCA1, ABCG1, LXRA, CPY27A1, and PPARγ) would be associated with cholesterol efflux capacity. Further, we hypothesized exposure to VAT EVs from patients with obesity would reduce macrophage cholesterol efflux capacity and cholesterol gene expression in vitro.

## Methods

### Subjects

Adolescent females and males (ages 12–19) with obesity (BMI > 25 kg/m^2^) or determined to be of Lean body composition (BMI ≤ 25) were recruited for this study. All subjects were enrolled prior to scheduled abdominal surgeries. Subjects with obesity completed a protein-sparing modified fast (~ 1000 kcal/day; 50–60 g protein) for 2 weeks prior to their bariatric surgery date. All Subjects completed an overnight fast prior to surgery and tissue collection. Detailed methodology is provided in Additional file 1: Methods.

#### Lipoprotein measurement

Lipoprotein particle concentration and diameters were quantified using the automated NMR approach [19]. Lipoprotein insulin resistance index (LPIR) was calculated as described previously [20]. Summary anthropometric and lipoprotein particle concentration and diameters data is listed in Table 1.

**Table 1:** Patient clinical characteristics

	n	Median (IQR)	p value*
Anthropometric			
Sex (F, M)	93	71, 22	–
Age (year)	93	17 (3)	0.04
Height (cm)	93	164 (13)	0.3
Weight (kg)	93	126 (45)	0.36
BMI (kg/m^2^)	93	45.2 (13.2)	0.38
HDL function			
Cholesterol efflux capacity	69	0.85 (0.17)	–
Lipid panel			
Total cholesterol	69	127 (34)	0.89
LDL-C, mg/dL	69	77 (38)	0.86
HDL-C, mg/dL	69	40 (13)	0.11
Triglycerides	69	56 (36)	0.48
NMR spectroscopy			
LDL particle concentration, nmol/L	69	933 (453)	0.15
HDL particle concentration, µmol/L	69	25.6 (5.3)	0.46
VLDL particle concentration, nmol/L	69	28.4 (20.4)	0.09
LDL particle size, nm	69	20.5 (9)	< 0.01
HDL particle size, nm	69	9.2 (0.7)	0.01
VLDL particle size, nm	69	48.4 (8.35)	0.17
Metabolic assessment			
Lipoprotein insulin resistance score	69	46 (25)	0.45
Inflammation assessment			
GlycA	69	429 (90)	0.15

Data are presented as Median (IQR). *p* value for Pearson product moment correlation coefficient for cholesterol efflux capacity

#### Cholesterol efflux capacity using ApoB depleted subject serum

Subject serum, collected prior to surgical procedures, was used to quantify cholesterol efflux capacity as previously described [11, 12, 21–23]. Liquid scintillation counting was employed to quantify efflux of radioactive a cholesterol from J774 cells. Quantity of radioactive cholesterol incorporated into cellular lipids was determined by means of isopropanol extraction from control wells not used in serum experiments. Efflux was calculated by the following formula: [(microcuries of 3H-cholesterol in mediums containing 2.8% apolipoprotein B-depleted serum-microcuries of 3H-cholesterol in serum-free mediums)/microcuries of 3H-cholesterol in cells extracted before the efflux step] × 100 [12].

### Circulating adipocyte-derived EVs microRNA profiles

#### Isolation of circulating adipocyte-derived EVs and microRNA profiles

Adipocyte-derived EVs were isolated using the commercially available EoxQuick Precipitation Solution (System Biosciences, Mountain View, CA) from the serum of an all-female subset, chosen to be phenotypically representative of the larger cohort, as previously described [15]. Total RNA was extracted from adipocyte-derived EVs using the commercially available SeraMir Exosome RNA Amplification Kit (System Biosciences, Mountain View, CA) according to manufacturer instructions. RNA was labeled with Affymetrix^®^ FlashTag™ Biotin HSR RNA Labeling Kit (Affymetrix, Santa Clara, CA) according to standard procedures. Labeled RNA was hybridized to Affymetrix GeneChip microRNA 4.0 arrays and run using a Fluidics Station 450 Protocol (FS450_002) (Affymetrix, Santa Clara, CA). MicroRNAs and ProbeIDs used for statistical analysis are provided in Additional file 2: Table S1 (Accession #: GSE125494).

### THP-1 macrophages and adipose-derived EVs incubation experiments

#### Extracellular vesicle isolation from visceral adipose tissue

Visceral adipose tissue, collected during abdominal surgeries, was promptly cultured using a previously published protocol [13, 24]. EV were isolated using the commercially available ExoQuick-TC Precipitation Solution. Previous studies by our group has demonstrated ~ 99% of EV isolated in this preparation are positive for adipocyte differentiation marker FABP4 [13].

#### Culturing of THP-1 cells

THP-1 human monocytes (American Type Culture Collection, Rockville, MD) were grown at 37 °C in a 5% CO_2_ atmosphere in RPMI-1640 (Invitrogen, Carlsbad, CA) supplemented with 10% fetal calf serum (FCS), penicillin, and streptomycin. To facilitate differentiation into macrophages, THP-1 cells were exposed to with 100 nM Phorbol 12-myristate 13-acetate (PMA) (Sigma-Aldrich, St. Louis, MO) for 24 h at 37 °C then the PMA-containing medium was replaced with complete RPMI-1640 supplemented. For EV exposure experiments, THP-1 cells were exposed to adipocyte-derived exosomes at 1 µg/mL, 3 µg/mL, or supplemented RPMI 1640 medium alone for 18 h at 37 °C.

#### THP-1 cholesterol uptake

Adipocyte-derived EVs were fluorescently-labeled with the Cytoplasmic Membrane Staining Kit (PromoKine, Heidelberg, Germany) and suspended in exosome-depleted FCS at a final concentration of 1 µg/mL or 3 µg/mL. THP-1 macrophages were incubated with fluorescently-labeled adipocyte-derived EVs only, 1,1′-dioctadecyl-3,3,3′,3′-tetramethylin docarbocyaninet (DiI)-oxLDL (Kalen Biomedical, Germantown, MD, USA) 5 µg/mL only, or both adipocyte-derived EVs and DiI-oxLDL for 4 h. Slides were fixed using 4% paraformaldehyde prepared using Vectashield mounting medium containing DAPI stain (Vector Laboratories, Inc., Burlingame, CA).

#### THP-1 cholesterol efflux assay

Cholesterol efflux was analyzed on THP-1 cells plated in 96 well plates at 1 × 10^6^ cells/mL in the presence of adipocyte-derived exosomes at 1 µg/mL, 3 µg/mL, or supplemented RPMI 1640 medium alone using the Amplex Red Cholesterol Assay kit (Molecular Probes, Eugene, OR), according to the manufacturer’s protocol.

#### Extraction of RNA and qRT-PCR

Immediately after the incubation period, total RNA was isolated with TRIzol (Thermo Fisher Scientific; Waltham, MA, USA) at 10^6^ cells/mL. 1 μg of total RNA was used to generate cDNA (murine leukemia virus reverse transcriptase (Applied Biosystems, Foster City, CA, USA). Equal amounts of cDNA were taken from each RT reaction mixture for PCR amplification using specific primers for ABCA1, ABCG1, CYP27A1, PPARγ, and LXRα (Table 2). qRT-PCR analysis was performed using a SYBR Green Reagent Kit according to the manufacturer’s instructions on the Roche Light Cycler 480 (Roche Applied Science, Penzburg, Germany). The C_T_ value for each gene was normalized to that of glyceraldehyde-3-phosphate dehydrogenase (GAPDH) and the relative expression level was calculated as the mean value of the unexposed to THP-1 as 1.

**Table 2:** RT-PCR primers

GAPDH	F 5′-ACCATCATCCCTGCCTCTAC-3′
	R 5′-CCTGTTGCTGTAGCCAAAT-3′
ABCA1	F 5′-GAAGTACATCAGAACATGGGC-3′
	R 5′-GATCAAAGCCATGGCTGTAG-3′
ABCG1	F 5′-CAGGAAGATTAGACACTGTGG-3′
	R 5′-GAAAGGGGAATGGAGAGAAG-3′
CYP27A1	F 5′-AAGCGATACCTGGATGGTTG-3′
	R 5′-TGTTGGATGTCGTGTCCACT-3′
PPARG	F 5′-CGACTGGGGATGTCTCATAATGC-3′
	R 5′-CAGGGGGGTGATGTGTTTGAA-3′
LXRA	F 5′-GGGGCCAGCCCCCAAAATGCTG-3′
	R 5′-GCATCCGTGGGAACATCAGTCG-3′

### Data analyses

Normality of data was assessed with the Shapiro–Wilk test and visualization of the distribution. If data were non-normally distributed, the data were log_2_-transformed and reassessed for normality. Relationship between anthropometric measures, traditional risk factors and cholesterol efflux were explored with Spearman’s rank correlation coefficient. To leverage the intersubject variability in cholesterol efflux capacity, subjects were clustered into groups using cholesterol efflux capacity via K-means cluster analysis. Multiple models of cluster analysis were analyzed utilizing cluster groups (*k*) of two through five. The goal was to identify the appropriate clustering to achieve the minimal average cluster center within clusters while maximizing distance between the separate cluster centers while enhancing statistical power to detect difference between groups. Our analysis identified three cluster groups (Additional file 2: Table S2) were the most appropriate and we have labeled these cluster groups: HIGH, Moderate (MOD), and LOW efflux capacity. With this methodology, our analysis has > 80% power to detect statistical differences between groups efflux capacity groups for NMR data. Statistical analysis was performed on the commercially available software OriginLab Pro 9.1 (OriginLab Corp.; Northampton, MA). NMR and anthropometric data were analyzed by one-way ANOVA with a Tukey’s honest significant difference post hoc test for intergroup differences in all the variables. Data that could not be normalized by log_2_-transformation were analyzed with a Kruskal–Wallis ANOVA and are denoted as such. For cell culture experiments, a two-way ANOVA (incubation × group) was used to test differences between exposure of adipose derived EVs at 1 μg/mL and 3 μg/mL and between EVs from subjects with obesity and Lean subjects. Significance was set a priori as p < 0.05. Tukey’s Honest Significant Difference post hoc test for intergroup differences in all analyses. To test for significant associations between subject cholesterol efflux capacity and circulating adipose-derived EVs microRNAs we utilized forward selection multivariate stepwise regression analysis. Unstandardized beta coefficients, 95% confidence intervals, and correlation coefficients are reported herein.

## Results

### Subject clinical and anthropometric data

The cohort of adolescent females (n = 93) with and without obesity had BMI ranging from 22 to 70 kg/m^2^ (Median [IQR] = 46.1 [35.0, 57.2]). All subjects identified as obese (n = 78, 47.0 [40.3, 70.5]) by BMI were > 99th percentile for age-adjusted BMI and all subjects identified as Lean (n = 15, 22.0 [19.5, 23.9]) were < 85th percentile. Subject clinical and anthropometric data is presented in Table 1.

### Cholesterol efflux capacity as a function of clinical and anthropometric variables

Cholesterol efflux capacity (n = 69, 0.86 [0.76, 0.94]), from J774A.1 cells, was measured in subject’s. Increasing age (r = 0.24, p = 0.04), LDL particle size (r = 0.33, p = 0.005), and HDL particle size (r = 0.30, p = 0.01) were significantly related to cholesterol efflux capacity in the overall cohort. Traditional ASCVD risk factors such as BMI (r = − 0.01, p = 0.9), HDL (r = 0.19, p = 0.11), LDL (r = 0.02, p = 0.83), total cholesterol (r = 0.02, p = 0.88), triglycerides (r = − 0.06, p = 0.62) did not correlate with cholesterol efflux capacity (Table 1). Measures of systemic inflammation (GlycA, r = − 0.17, p = 0.16) and insulin resistance (LPIR, r = − 0.09, p = 0.45) were also not associated.

**Fig. 1:**
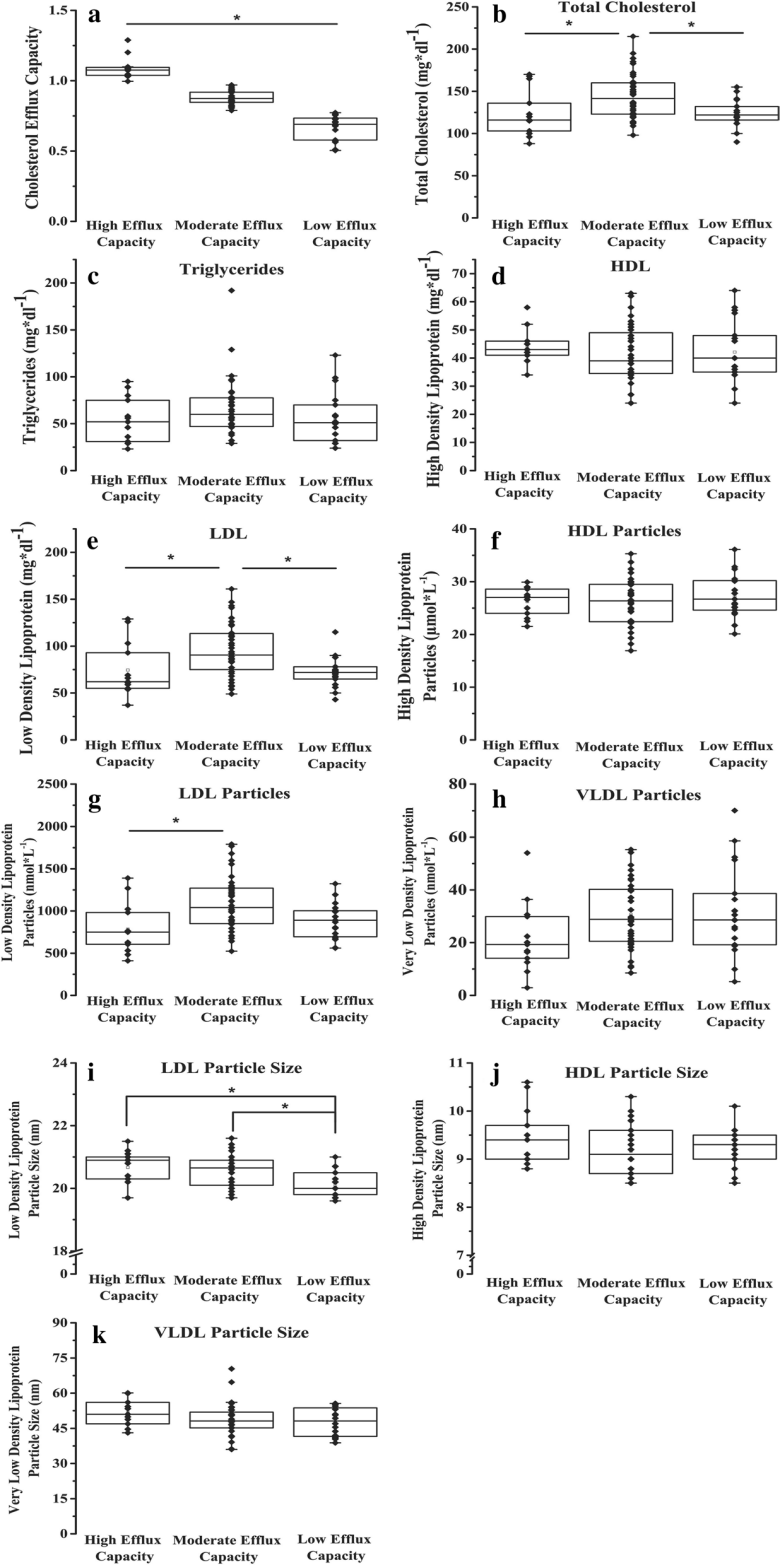
Serum lipoprotein particle profiles of HIGH (n = 13), MOD (n = 69), and LOW (n = 19) Cholesterol efflux capacity groups. **a** Cholesterol efflux capacity groups as determined by k-means cluster analysis; *p < 0.001 between groups. **b** Total cholesterol; *p < 0.05 MOD vs. HIGH and MOD vs. LOW. **c** Total Triglycerides. **d** High density lipoprotein concentration. **e** Low density lipoprotein concentration; *p < 0.05 MOD vs. HIGH and MOD vs. Low. **f** High density Lipoprotein particle concentration. **g** Low density lipoprotein particle concentration; *p < 0.05 MOD vs. HIGH. **h** Very low density lipoprotein particle concentration. **i** Low density lipoprotein particle size; *p < 0.05 HIGH vs. LOW and MOD vs. LOW. **j** High density lipoprotein particle size. **k** Very low-density lipoprotein particle size

**Fig. 2:**
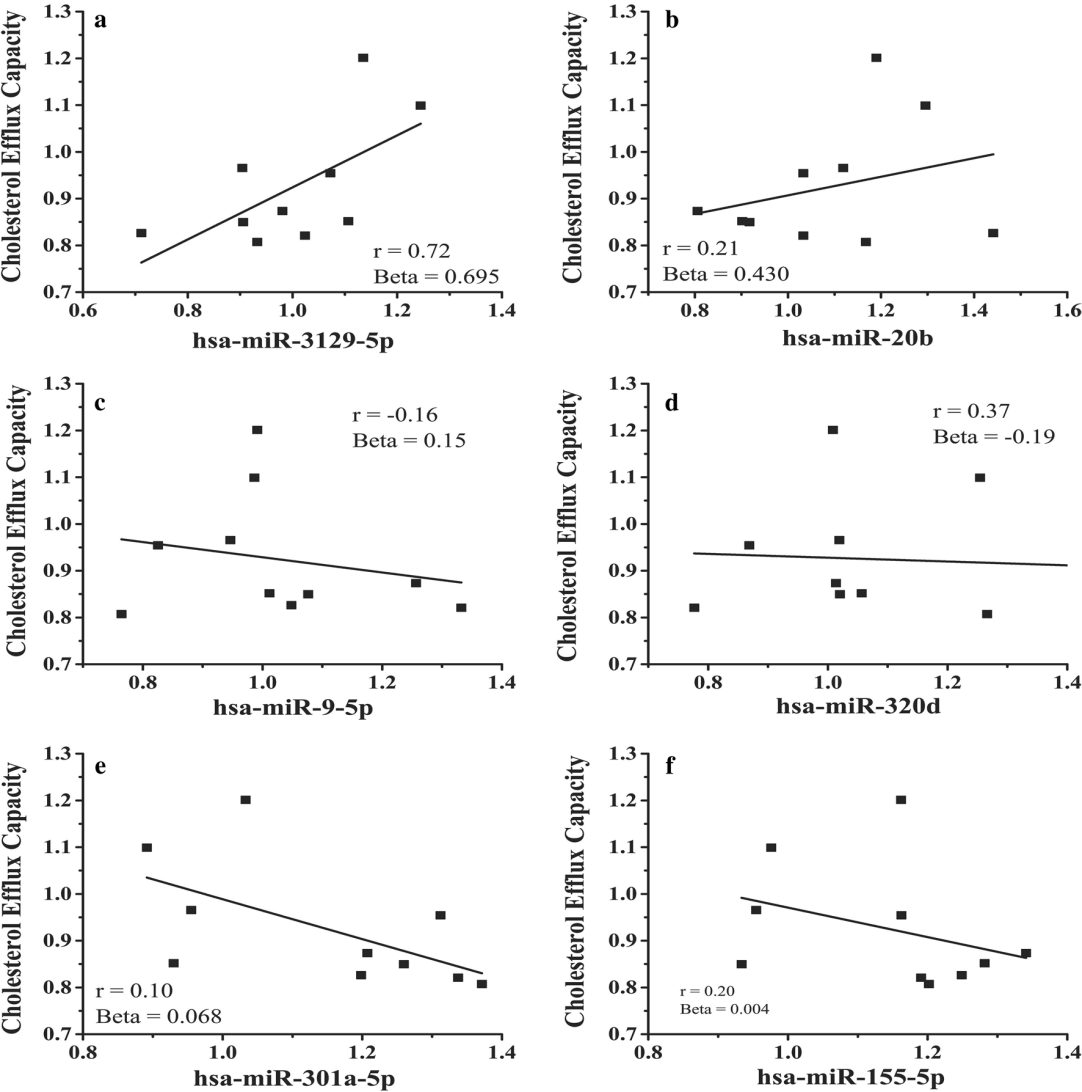
Significant microRNAs from circulating adipocyte-derived EVs microRNAs identified via multivariate analysis. Identified microRNAs include (**a**) miR-3129-5p  (**b**) miR-20b, (**c**) miR9-5p, (**d**) miR-320d, (**e**) miR301a-5p, (**f**) miR-155-5p. Pearson’s correlation coefficient (r) are provided as an indicator of the relationship between the individual microRNA and cholesterol efflux

Subjects were then clustered based on cholesterol efflux capacity into HIGH (n = 13, 1.07 [1.04, 1.09]), MOD (n = 36, 0.87 [0.85, 0.92]), and LOW (n = 19, 0.69 [0.57, 0.73]) via K-means cluster analysis (Fig. 1a). K-Means cluster analysis statistics are available in Additional file 2: Table S2. Subjects in the HIGH cholesterol efflux capacity cluster were older (Age = 18 [17, 20], p = 0.03) as compared to the MOD (17 [15, 18]) and LOW (16 [15, 17]) clusters. Post-hoc analyses showed that the MOD cluster had significantly higher total cholesterol (TC = 142 [123, 160], p = 0.002, Fig. 1b) and low-density lipoprotein concentration (LDL = 75 [49, 91], p = 0.01, Fig. 2e) compared to both the HIGH (TC = 116 [103, 136], LDL = 62 [55, 93] and LOW (TC = 122 [116, 132], LDL = 72 [65, 78]) clusters. The MOD (LDL-p = 523 [523, 1042]) cluster had a significantly (p = 0.002, Fig. 1g) higher LDL particle concentration than the HIGH (606 [411, 750]) cluster. Furthermore, the HIGH (LDL-z = 20.3 [19.7, 20.9], p = 0.007) and MOD (LDL-z = 20.1 [19.7, 20.7], p = 0.003) clusters had larger LDL particle size than the LOW (19.8 [19.6, 20] cluster (Fig. 1i).

### Cholesterol efflux capacity as a function of circulating adipocyte-derived EV microRNAs

We isolated adipocyte-derived EVs from a subset, selected to be representative of the larger cohort, of subjects’ serum with (n = 8, age = 17 ± 3, BMI = 52.8 ± 9.6, cholesterol efflux = 0.89 ± 0.10) and without obesity (n = 3, age = 18 ± 3, BMI = 23.1 ± 1.2, Cholesterol Efflux = 0.99 ± 0.20). These subjects were representative of our cohort for cholesterol efflux (p = 0.14), BMI (p = 0.29), and age (p = 0.36). We limited our analyses to 89 microRNAs, identified from our filtering protocol described in Additional file 1: Methods, that had previously established or highly predicted interaction with well-known cholesterol transport mRNAs: ABCA1, ABCG1, CYP27A1, PPARγ, and LXRα. Multivariate analyses identified seven (Fig. 2a–f) microRNAs associated with cholesterol efflux capacity: (Fig. 2a) miR-3129-5p (Beta = 0.695, 95% CI 0.694 to 0.696), (Fig. 2b) miR-20b (0.430, 0.429 to 0.431), (Fig. 2c) miR9-5p (0.111, 0.110 to 0.112), (Fig. 2d) miR-320d (− 0.190, − 0.191 to − 0.189), (Fig. 2e) miR301a-5p (0.042, 0.041 to 0.043), (Fig. 2f) miR-155-5p (0.004, 0.004 to 0.005). Notably, all significant microRNAs targeted ABCA1.

### In vitro macrophage cholesterol efflux

To test if adipocyte-derived EVs from VAT alter macrophage cholesterol efflux we incubated THP-1 macrophages with EVs isolated from surgically acquired VAT. EVs were isolated from subjects with (n = 15, age = 16 ± 2, BMI = 44.8 ± 7.2) and without (n = 12, age = 15 ± 4, BMI = 21.6 ± 3.4) obesity. Subjects were selected to be representative of our larger cohort and on the availability of VAT explants for EV isolation.

**Fig. 3:**
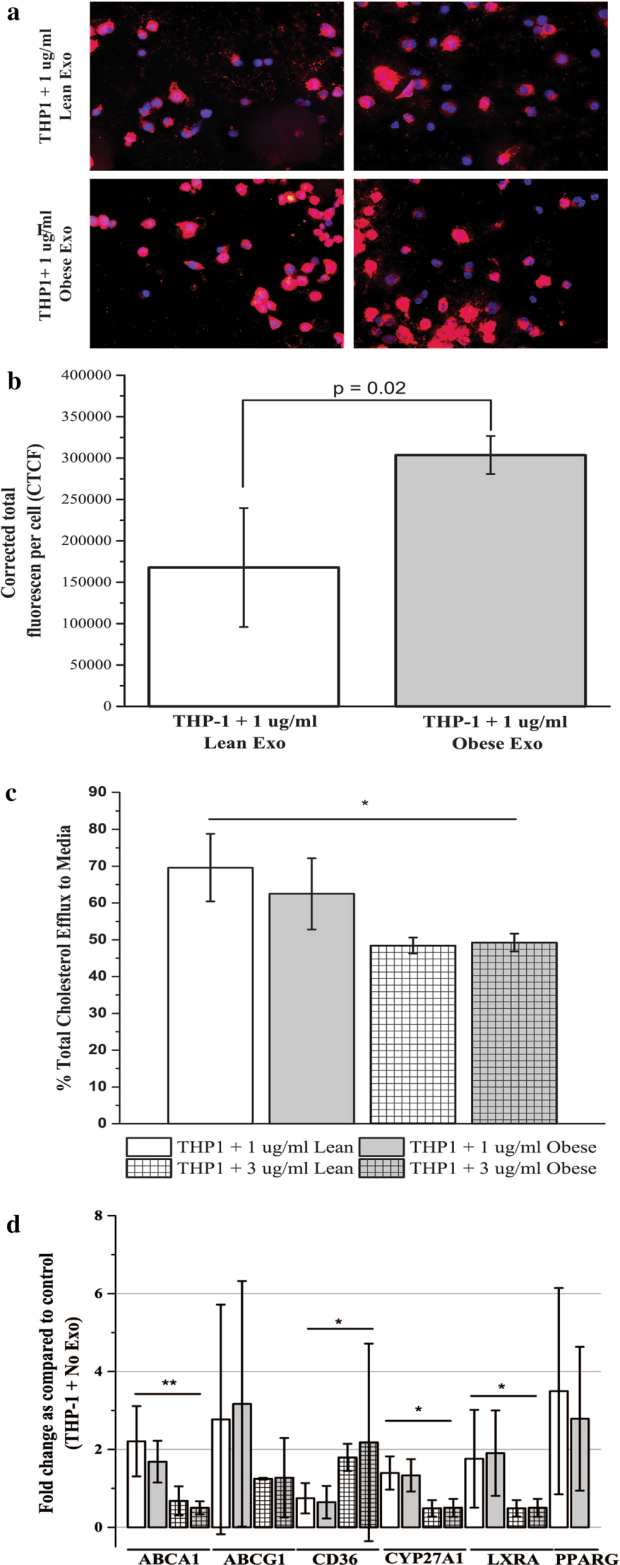
Effect on THP-1 cells of adipocyte-derived EVs from subjects with Obesity and Lean subjects on cholesterol efflux gene expression and cholesterol efflux to media. THP-1 cells were incubated with obese and Lean visceral adipocyte-derived EVs for 24 h. **a** Images of fluorescently labeled THP-1 (blue) macrophages, fluorescently labeled Dil-oxLDL (red), and exosomes (1 μg/mL; unlabeled) VAT tissue exosomes from subjects with Obesity and Lean subjects. **b** Dil-oxLDL uptake by THP-1 cells is significantly increased (81%, p = 0.02) when incubated with adipocyte-derived EVs from obese subjects as compared exosomes from Lean subjects. Data is presented as mean ± std of 1 μg/mL experiments with n = 5 for each group at 1 μg/mL of EVs. **c** Cholesterol concentration was detected by fluorometric assay in both THP-1 cells and the surrounding medium after 24 h exposure to adipocyte-derived EVs from obese subjects and from Lean subjects. *p < 0.05 for experiments with 3 μg/mL exosomes vs 1 μg/mL exosomes. **d** ABCA1, ABCG1, CD36, 27OH, LXRA, and PPARG, measured in THP-1 cells incubated with adipocyte-derived EVs from obese and Lean subjects using qRT-PCR. Data is presented at mean ± std of fold changes in comparison to THP-1 cells not exposed (Control); **p < 0.01 and *p < 0.01 for experiments with 3 μg/mL exosomes vs 1 μg/mL EVs

First, we examined the formation of macrophage-derived foam cells when exposed to EV from obese and Lean subjects. THP-1 macrophages were incubated with 1 µg/mL exosomes and Dil-oxLDL. Exposure to EVs from obese subjects increased THP-1 Dil-oxLDL uptake (Fig. 3a, b) by 81% (p = 0.02) in comparison to exposure to EVs from Lean subjects. Cholesterol efflux from THP-1 macrophages (Fig. 3c) was significantly (p < 0.001) reduced when exposed to VAT EV at 3 μg/mL (49% ± 2%; normalized to no EV control) as compared to 1 μg/mL (66% ± 10%). There was no difference (p = 0.44) between incubations with VAT EV from subjects with and without obesity.

Next, we focused on cholesterol efflux gene expression (i.e. ABCA1, ABCG1, CYP27A1, PPARγ, and LXRα; Table 2) in THP-1 cells exposed to the EVs. All experiments were analyzed as a fold-change to untreated control wells. When exposed to VAT EVs at 3 μg/mL, ABCA1 (FC = 0.5 ± 0.2 vs. 1.9 ± 0.8; p < 0.001), CD36 (2.1 ± 0.8 vs. 0.7 ± 0.4, p = 0.02), CYP27A1 (0.9 ± 0.5 vs. 1.4 ± 0.4), and LXRA (0.5 ± 0.2 vs. 1.8 ± 1.1) were differentially expressed in comparison to exposure to VAT EVs at 1 μg/mL. No differences were detected when comparing exposure to VAT EVs subjects with and without obesity.

## Discussion

In this study we show, for the first time, significant alterations in cholesterol efflux capacity in adolescents throughout the range of BMI, a relationship between six circulating adipocyte-derived EVs microRNAs targeting ABCA1 and cholesterol efflux capacity, and in vitro alterations of cholesterol efflux in THP-1 macrophages exposed to VAT adipocyte-derive EVs acquired from human subjects. These results suggest that adipocyte-derived EVs, and their microRNA content, may play a critical role in the early pathological development of ASCVD.

ASCVD remains the leading cause of morbidity and mortality worldwide [1]. Although primarily a disease of adults, youth with obesity show evidence of subclinical ASCVD [2–4], which places them at increased risk as adults for coronary heart disease [3] and stroke [5]. Primary prevention of ASCVD would be informed by better understanding of the early pathologic events in youth with obesity. One of the hallmarks of ASCVD is macrophage cholesterol efflux [11, 12, 21–23, 25] impairment which leads to intracellular accumulation of modified LDL and subsequent generation of plaque-forming lipid-rich foam cells [6]. This is the first study demonstrating a wide range of cholesterol efflux capacity in adolescents throughout the BMI continuum (BMI range for study: 22–70 kg/m^2^). By using cluster analysis, we show that differences in efflux capacity are not related to differences in BMI, systemic inflammation (GlycA), or insulin resistance (LPIR). Furthermore, these changes are occurring before any clinically detectable changes in traditional lipid parameters would suggest concern. The MOD (significant) and Low (non-significant) efflux capacity group did show higher total cholesterol, LDL, and LDL particle concentrations as compared to the High efflux capacity groups which may indicate alterations in efflux capacity are impacting circulating lipid profiles.

Adipose tissue can be considered a metabolic organ capable of communicating with cell types relevant to ASCVD, including macrophages [26]. More recently adipocyte-derived EVs have become of significant interest as a potential mechanism linking adipose tissue communication with other peripheral tissues. In obese mice, adipocyte-derived EVs contribute to the development of insulin resistance via activation of adipose-resident macrophages and secretion of pro-inflammatory cytokines that can result in insulin resistance [24]. Furthermore, they have been linked to macrophage polarization, foam cell formation, and aortic plaque deposits [18]. Thus, the effect of adipocyte-derived EVs on macrophage foam cell formation is an emerging area of interest, though the mechanism through which they cause disturbances is not well understood.

We focused on adipocyte-derived exosomal microRNAs for multiple reasons: (1) the accumulating evidence for the role of microRNAs in ASCVD [27]; (2) due to our previous work indicating a high amount of small non-coding RNAs in adipocyte-derived EVs as compared to other genetic and molecular material [13] and; (3) that adipose tissue is a significant source of circulating microRNAs [17]. This led us to hypothesize that adipocyte-derived EVs microRNAs would target mRNAs involved in macrophage cholesterol efflux. In our subset of adolescents with and without obesity we identified six adipocyte-derived EVs microRNAs (Fig. 2a–f, all targeting ABCA1, to be significantly related to cholesterol efflux capacity. ABCA1 is a well-studied regulator of macrophage cholesterol efflux, working to prevent excess intracellular cholesterol accumulation [28]. Further work is required to understand the role of these microRNA’s role, individually and in concert, in regulating ABCA1 expression in macrophages and resultant changes in macrophage cholesterol efflux.

To extend the work of adipocyte-derived exosomes in animal and cell models, we sought to establish that visceral adipocyte-derived EVs, isolated from VAT of our adolescent cohort, impair macrophage cholesterol efflux in THP-1 cells. THP-1 human monocytic leukemia cells were chosen for the study because they share many properties with normal human monocytes, including expression of scavenger receptors and cholesterol transport proteins, and are a well-accepted model for ASCVD [29]. Our present study is the first study to utilize human samples and supports a role for adipocyte-derived EV in cholesterol efflux impairment. Exposure of THP-1 macrophages to exosomes isolated from VAT from obese subjects significantly increased Dil-oxLDL retention and resulted in decreased cholesterol efflux in a dose-dependent manner. Furthermore, we show an EVs dose-dependent alteration of macrophage cholesterol efflux genes ABCA1, CD36, CYP27A1, and LXRA. Together these experiments help extend the animal work [18, 24] and provide the first evidence that EVs from human adipose tissue result in the dysregulation of cholesterol efflux in vitro.

Contrary to our original hypothesis, we do not show an effect of obesity on THP-1 macrophage cholesterol efflux. This is a similar finding to that of Xie et al. [18] who showed similar effects of VAT EVs from wild type mice and mice fed a high fat diet. Given our findings of circulating EV microRNAs targeting ABCA1, we suspect that EVs, in part, exert their pro-atherogenic effect through transfer of microRNAs. However, our in vitro experiments cannot rule out other potential exosomal mechanisms such as macrophage polarization or protein signaling [18]. More studies testing various conditions are needed to fully elucidate how adipose-derived EVs regulate macrophage function or interact with other molecules, such as ox-LDL, to influence macrophage function. We also limited our studies to only using THP-1 cells, which is a limitation that future studies should address by using multiple cell lines, including primary monocyte derived macrophages. Further studies exploring the role of specific exosomal microRNAs are needed to help elucidate the connection between circulating EVs microRNAs, macrophage behavior, and macrophage cholesterol efflux. More studies using EVs isolated from human adipose tissue, as well as other significant sources of EVs such as platelets and skeletal muscle, are needed as human obesity is a multifactorial and heterogenous condition not easily captured in animal models.

Obesity, and specifically the accumulation of visceral adipose tissue, is a significant risk factor in the development of chronic cardiometabolic and increased cardiovascular risk profiles [3, 4]. However, the molecular link between visceral adipose tissue and peripheral tissue dysfunction is still poorly understood. More recent thinking has moved away from focusing solely on the quantity of adiposity, but instead understanding the molecular changes in adipose tissue that may drive these multifactorial diseases [6]. Our group has focused on adipocyte-derived EVs and previously demonstrated obesity-driven changes in adipocyte-derived EVs microRNAs and changes following bariatric weight-loss surgery [13, 15]. MicroRNAs, and specifically microRNAs packaged in EVs, are ideal for tissue crosstalk due to the stable nature of microRNAs and the cellular access the lipid vesicle provides [17, 27]. Furthermore, adipocyte-derived EV microRNAs offer a potential biomarker to determine the molecular nature of the adiposity and risk for developing cardiovascular disease and comorbidities. Our data on the relationship between adipocyte-derived EV microRNAs and cholesterol efflux capacity, as well as the in vitro alterations of macrophage cholesterol efflux, offer potential starting points for further mechanistic and longitudinal studies.

## Conclusion

Our study shows evidence that cholesterol efflux capacity alterations may, in part, be driven by adipocyte-derived EV microRNAs. Alterations in cholesterol efflux capacity of adolescents is not related to BMI, systemic inflammation, or insulin resistance and occur before major changes in lipid profiles. We identified six microRNAs in the circulation, packaged in adipocyte-derived EVs, that target ABCA1 and are significantly associated with cholesterol efflux capacity in the adolescent with and without obesity. Furthermore, in vitro exposure of THP-1 macrophages to adipocyte-derived exosomes from VAT altered cholesterol efflux and cholesterol efflux gene expression. Dil-oxLDL uptake was the only measure affected by the obesity status of the EV donor. These findings are the first studies to use adipocyte-derived EVs from human subjects and add to the growing evidence that adipocyte-derived EVs are a significant factor in tissue cross-talk and may, in part, drive the pathological events leading to cardiometabolic diseases.

## Additional files

**Additional file 1.** Additional Methods.

**Additional file 2: Table S1.** Mature Cholesterol Efflux MicroRNA for Multivariate Analysis. **Table S2.** K-Means Cluster Statistics.

## Data Availability

Affymetrix GeneChip 4.0 microRNA profiles used in this study are publicly available in the Gene Expression Omnibus (Accession #: GSE125494). Other data, to the extent reasonable for the protection of Private Health Information, may be available upon request.

## References

[1] Messiah SE, Vidot DC, Gurnurkar S, Alhezayen R, Natale RA, Arheart KL (2014). Obesity is significantly associated with cardiovascular disease risk factors in 2- to 9-year-olds. J Clin Hypertens (Greenwich)..

[2] Kelly AS, Barlow SE, Rao G, Inge TH, Hayman LL, Steinberger J (2013). Severe obesity in children and adolescents: identification, associated health risks, and treatment approaches: a scientific statement from the American Heart Association. Circulation.

[3] Kelly AS, Metzig AM, Schwarzenberg SJ, Norris AL, Fox CK, Steinberger J (2012). Hyperleptinemia and hypoadiponectinemia in extreme pediatric obesity. Metab Syndr Relat Disord..

[4] Lawlor DA, Leon DA (2005). Association of body mass index and obesity measured in early childhood with risk of coronary heart disease and stroke in middle age: findings from the aberdeen children of the 1950s prospective cohort study. Circulation.

[5] Autieri MV (2016). Adipose inflammation at the heart of vascular disease. Clin Sci.

[6] Fuster JJ, Ouchi N, Gokce N, Walsh K (2016). Obesity-induced changes in adipose tissue microenvironment and their impact on cardiovascular disease. Circ Res.

[7] Deiuliis JA, Syed R, Duggineni D, Rutsky J, Rengasamy P, Zhang J (2016). Visceral adipose MicroRNA 223 is upregulated in human and murine obesity and modulates the inflammatory phenotype of macrophages. PLoS ONE.

[8] Ley K, Miller YI, Hedrick CC (2011). Monocyte and macrophage dynamics during atherogenesis. Arterioscler Thromb Vasc Biol.

[9] Voloshyna I, Godoy J, Littlefield M, Leon J, Magana M (2014). Advanced glycation end products promote pro-atherogenic changes in cholesterol transport: a possible mechanism for cardiovascular risk in diabetes. Intern Med S..

[10] Voloshyna I, Modayil S, Littlefield MJ, Belilos E, Belostocki K, Bonetti L (2013). Plasma from rheumatoid arthritis patients promotes pro-atherogenic cholesterol transport gene expression in THP-1 human macrophages. Exp Biol Med (Maywood)..

[11] Khera AV, Cuchel M, de la Llera-Moya M, Rodrigues A, Burke MF, Jafri K (2011). Cholesterol efflux capacity, high-density lipoprotein function, and atherosclerosis. N Engl J Med.

[12] Mehta NN, Li R, Krishnamoorthy P, Yu Y, Farver W, Rodrigues A (2012). Abnormal lipoprotein particles and cholesterol efflux capacity in patients with psoriasis. Atherosclerosis..

[13] Ferrante SC, Nadler EP, Pillai DK, Hubal MJ, Wang Z, Wang JM (2015). Adipocyte-derived exosomal miRNAs: a novel mechanism for obesity-related disease. Pediatr Res.

[14] Koeck ES, Iordanskaia T, Sevilla S, Ferrante SC, Hubal MJ, Freishtat RJ (2014). Adipocyte exosomes induce transforming growth factor beta pathway dysregulation in hepatocytes: a novel paradigm for obesity-related liver disease. J Surg Res.

[15] Hubal MJ, Nadler EP, Ferrante SC, Barberio MD, Suh JH, Wang J (2017). Circulating adipocyte-derived exosomal MicroRNAs associated with decreased insulin resistance after gastric bypass. Obesity (Silver Spring)..

[16] Valadi H, Ekstrom K, Bossios A, Sjostrand M, Lee JJ, Lotvall JO (2007). Exosome-mediated transfer of mRNAs and microRNAs is a novel mechanism of genetic exchange between cells. Nat Cell Biol.

[17] Thomou T, Mori MA, Dreyfuss JM, Konishi M, Sakaguchi M, Wolfrum C (2017). Adipose-derived circulating miRNAs regulate gene expression in other tissues. Nature.

[18] Xie Z, Wang X, Liu X, Du H, Sun C, Shao X (2018). Adipose-derived exosomes exert proatherogenic effects by regulating macrophage foam cell formation and polarization. J Am Heart Assoc..

[19] Otvos JD, Mora S, Shalaurova I, Greenland P, Mackey RH, Goff DC (2011). Clinical implications of discordance between low-density lipoprotein cholesterol and particle number. J Clin Lipidol..

[20] Shalaurova I, Connelly MA, Garvey WT, Otvos JD (2014). Lipoprotein insulin resistance index: a lipoprotein particle-derived measure of insulin resistance. Metab Syndr Relat Disord..

[21] Khera AV, Rader DJ (2009). Discovery and validation of new molecular targets in treating dyslipidemia: the role of human genetics. Trends Cardiovasc Med.

[22] Salahuddin T, Natarajan B, Playford MP, Joshi AA, Teague H, Masmoudi Y (2015). Cholesterol efflux capacity in humans with psoriasis is inversely related to non-calcified burden of coronary atherosclerosis. Eur Heart J.

[23] Lerman JB, Joshi AA, Chaturvedi A, Aberra TM, Dey AK, Rodante JA (2017). Coronary plaque characterization in psoriasis reveals high-risk features that improve after treatment in a prospective observational study. Circulation.

[24] Deng ZB, Poliakov A, Hardy RW, Clements R, Liu C, Liu Y (2009). Adipose tissue exosome-like vesicles mediate activation of macrophage-induced insulin resistance. Diabetes.

[25] Voloshyna I, Reiss AB (2011). The ABC transporters in lipid flux and atherosclerosis. Prog Lipid Res.

[26] Choe SS, Huh JY, Hwang IJ, Kim JI, Kim JB (2016). Adipose tissue remodeling: its role in energy metabolism and metabolic disorders. Front Endocrinol (Lausanne)..

[27] Feinberg MW, Moore KJ (2016). MicroRNA regulation of atherosclerosis. Circ Res.

[28] Yvan-Charvet L, Wang N, Tall AR (2010). Role of HDL, ABCA1, and ABCG1 transporters in cholesterol efflux and immune responses. Arterioscler Thromb Vasc Biol.

[29] Qin Z (2012). The use of THP-1 cells as a model for mimicking the function and regulation of monocytes and macrophages in the vasculature. Atherosclerosis..

