# Supplementary material for: Cholesterol efflux alterations in adolescent obesity: role of adipose-derived extracellular vesical microRNAs
Source: J Transl Med. 2019 Jul 22;17:232. doi: 10.1186/s12967-019-1980-6 (PMC6647309; doi:10.1186/s12967-019-1980-6)
Supplement: Supplementary file 1 — Additional file 1. Additional Methods. [file 12967_2019_1980_MOESM1_ESM.docx]

**Additional Methods**

*Lipoprotein* Measurement: Briefly, subjects are scored based on very low-density lipoprotein (VLDL) size, large VLDL concentrations, low density lipoprotein (LDL) size, small LDL concentrations, High density lipoprotein (HDL) size, and large HDL concentration. Values for each variable are weighted and summated to produce a final LPIR score, with high values representing increased risk for cardiometabolic diseases.

*Cholesterol Efflux Capacity Using ApoB Depleted Subject Serum:* The murine macrophage cell line J774 were plated and radiolabeled with 2 µCi of 3-H cholesterol per milliliter. To induced upregulation of ABCA1, cells were incubated with 0.3 mM 8-(4-chlorophenylthio)-cyclic AMP for 6 hours. Efflux medium containing 2.8% apolipoprotein B-depleted serum were added for 4 hours. Apo-B-depleted serum was prepared by adding 40 parts polyethylene glycol solution (PEG; 20% PEG 8000 MW in 200 mM glycine buffer, pH 7.4) was added to 100 parts serum and mixed by pipetting. Samples were incubated at room temperature for 20 minutes before microcentrifugation at 10,000 rpm for 30 minutes at 4 °C. Apo-B-containing lipoproteins are pelleted by this procedure. The supernatant, containing the HDL fraction, was then diluted in 14 mM MEM-HEPES (no bicarbonate) + 0.15 mM cAMP to 2.8% (~2% serum). All steps were performed with acyl-coenzyme A:cholesterol acyltransferase inhibitor CP113,818 (2 µg per ml) present.

*Isolation of Circulating Adipocyte-derived Extracellular vesicles and microRNA Profiles*: Briefly, subjects’ serum samples were centrifuged at 3,000 x g for 15 minutes to remove cellular debris and the supernatant filtered through 22 µm syringe filter (Sarstedt, Nümbrecht, Germany) before addition of the commercially available ExoQuick Precipitation Solution (System Biosciences, Mountain View, CA). To isolate adipocyte-derived extracellular vesicles from total extracellular vesicles in PBS, fatty acid binding protein 4 (FABP4) was used as a sensitive and specific marker for adipocyte-derived extracellular vesicles from ^1, 2^. We used commercially-available antibody complexes and dextran-coated magnetic particles (StemCell Technologies, Vancouver, BC, Canada) to target FABP4+ extracellular vesicles in plasma.

Resulting data were analyzed in Expression Console using RMA+DMBG (Affymetrix). Only mature human microRNAs were retained for statistical purposes. For this study, we utilized Ingenuity Pathway Analysis’ (IPA; Qiagen; Germantown, MD) microRNA Target Filter to identify microRNAs targeting (experimentally validated or highly predicted) well known cholesterol efflux related genes: ABCA1, ABCG1, CYP27A1, PPARG, and LXRA. IPA Target Filter utilizes TargetScan, TarBase, miRecords, and The Ingenuity Knowledge Base to match microRNAs with target mRNAs. To confirm our IPA Target Filter results, miRTarVis (a novel visual analytics tool for integrated analyses of microRNA and mRNA expression profiles with microRNA target prediction algorithms) was used ^3^.

*Exosome Isolation from Visceral Adipose Tissue:* Adipocyte-derived extracellular vesicles were isolated from culture supernatants using ExoQuick-TC Precipitation Solution (System Biosciences, Mountain View, CA) and filtered through a 22 µm syringe. Appropriate volumes of ExoQuick-TC were added to the supernantant and incubated overnight at 4 °C. Samples were centrifuged for 30 minutes at 1500 x g and the supernatant discarded. The exosome pellet was resuspended to the appropriate concentration using media as described below.

*THP-1 Cholesterol Uptake:* Microscopic images were obtained using a Nikon A1 microscopy unit at 40X magnification and a DS-Ri1 digital camera. Accumulation of adipocyte-derived extracellular vesicles and/or DiI-oxLDL in cells was determined by fluorescent intensity, quantified from at least three random fields (1024 x 1024 pixels) per slide, from three slides per experimental condition.

*Extraction of RNA and qRT-PCR*: Briefly, cells were lysed in TRIzol reagent and RNA was precipitated with isopropanol before being pelleted. The pellet was washed in 75% ethanol. RNA was solubilized in RNase-free water and incubated at 60°C for 15 minutes. RNA quantity and quality was assessed using a Hitachi U2010 (Hitachi Ltd.; Tokyo, Japan) spectrophotometer. Samples with a 260:280 nm ratio > 1.8 were used for qRT-PCR. cDNA was copied from 1 µg of total RNA using Moloney murine leukemia virus reverse transcriptase (Applied Biosystems, Foster City, CA, USA) primed with oligo dT (Applied Biosystems). PCR products were measured using Roche Applied Science software, provided by the manufacturer. Fluorescence emission spectra were monitored and analyzed. PCR products were measured by the threshold cycles (C_T_), at which specific fluorescence becomes detectable. Non-template controls were included for each primer pair to check for significant levels of contamination. A melting-curve analysis was performed to assess the specificity of the amplified PCR products.

References

[1] Shan, T, Liu, W and Kuang, S, Fatty acid binding protein 4 expression marks a population of adipocyte progenitors in white and brown adipose tissues, FASEB 2013;27:277-287.

[2] Hubal, MJ, Nadler, EP, Ferrante, SC, et al., Circulating adipocyte-derived exosomal MicroRNAs associated with decreased insulin resistance after gastric bypass, Obesity (Silver Spring), 2017;25:102-110.

[3] Jung, D, Kim, B, Freishtat, RJ, et al., miRTarVis: an interactive visual analysis tool for microRNA-mRNA expression profile data, BMC Proceedings 2015:S2.
