# Supplementary material for: Cholesterol efflux alterations in adolescent obesity: role of adipose-derived extracellular vesical microRNAs
Source: J Transl Med. 2019 Jul 22;17:232. doi: 10.1186/s12967-019-1980-6 (PMC6647309; doi:10.1186/s12967-019-1980-6)
Supplement: Supplementary file 2 — Additional file 2: Table S1. Mature Cholesterol Efflux MicroRNA for Multivariate Analysis. Table S2. K-Means Cluster Statistics. [file 12967_2019_1980_MOESM2_ESM.docx]

**Additional Tables**

| **Table S1.** Mature Cholesterol Efflux MicroRNA for Multivariate Analysis | |  |  |  |  |
| --- | --- | --- | --- | --- | --- |
| \| hsa-miR-1-3p \| hsa-miR-185-5p \| hsa-miR-27a-3p \| hsa-miR-369-3p \| hsa-miR-526b-3p \| \| --- \| --- \| --- \| --- \| --- \| \| hsa-miR-101-3p \| hsa-miR-199a-3p \| hsa-miR-27b-3p \| hsa-miR-372-3p \| hsa-miR-5590-3p \| \| hsa-miR-106a-5p \| hsa-miR-199a-5p \| hsa-miR-301a-3p \| hsa-miR-373-3p \| hsa-miR-613 \| \| hsa-miR-124-3p \| hsa-miR-199b-3p \| hsa-miR-301b-3p \| hsa-miR-376c-3p \| hsa-miR-758-3p \| \| hsa-miR-1271-5p \| hsa-miR-199b-5p \| hsa-miR-302a-3p \| hsa-miR-384 \| hsa-miR-760 \| \| hsa-miR-128-3p \| hsa-miR-19a-3p \| hsa-miR-302b-3p \| hsa-miR-429 \| hsa-miR-9-5p \| \| hsa-miR-1297 \| hsa-miR-19b-3p \| hsa-miR-302c-3p \| hsa-miR-4295 \| hsa-miR-93-5p \| \| hsa-miR-130a-3p \| hsa-miR-200b-3p \| hsa-miR-302d-3p \| hsa-miR-4306 \| hsa-miR-96-5p \| \| hsa-miR-130b-3p \| hsa-miR-200c-3p \| hsa-miR-302e \| hsa-miR-4429 \|  \| \| hsa-miR-140-3p \| hsa-miR-206 \| hsa-miR-3129-5p \| hsa-miR-4465 \|  \| \| hsa-miR-140-5p \| hsa-miR-20a-5p \| hsa-miR-320a \| hsa-miR-454-3p \|  \| \| hsa-miR-142-5p \| hsa-miR-20b-5p \| hsa-miR-320b \| hsa-miR-4644 \|  \| \| hsa-miR-144-3p \| hsa-miR-216a-3p \| hsa-miR-320c \| hsa-miR-5195-3p \|  \| \| hsa-miR-145-5p \| hsa-miR-216b-5p \| hsa-miR-320d \| hsa-miR-519d-3p \|  \| \| hsa-miR-148a-3p \| hsa-miR-219a-2-3p \| hsa-miR-330-3p \| hsa-miR-520a-3p \|  \| \| hsa-miR-148b-3p \| hsa-miR-23a-3p \| hsa-miR-33a-5p \| hsa-miR-520b \|  \| \| hsa-miR-152-3p \| hsa-miR-23b-3p \| hsa-miR-33b-5p \| hsa-miR-520c-3p \|  \| \| hsa-miR-155-5p \| hsa-miR-23c \| hsa-miR-340-5p \| hsa-miR-520d-3p \|  \| \| hsa-miR-17-5p \| hsa-miR-26a-5p \| hsa-miR-3666 \| hsa-miR-520e \|  \| \| hsa-miR-183-5p \| hsa-miR-26b-5p \| hsa-miR-3681-3p \| hsa-miR-520f-3p \|  \| |  | |  |  |  |

**Table S2.** K-Means Cluster Statistics

| ***k* = 2** | n | Within Cluster Sum of Square | Average Distance | Maximum Distance | Distance Between Clusters | | | | | |
| --- | --- | --- | --- | --- | --- | --- | --- | --- | --- | --- |
| Cluster1 | 24 | 0.97134 | 0.15918 | 0.61408 | - | | | | | |
| Cluster2 | 45 | 1.31369 | 0.14017 | 0.52876 | .44989 | | | | | |
| ***k* =3** | n | Within Cluster Sum of Square | Average Distance | Maximum Distance | Distance Between Clusters | | | | | |
|  |  |  |  |  | Cluster 1 | | | | Cluster 2 | |
| Cluster1 | 13 | 0.38097 | 0.11562 | 0.47138 | - | | | | .4105 | |
| Cluster2 | 36 | 0.25237 | 0.06903 | 0.1695 | .41905 | | | | - | |
| Cluster3 | 20 | 0.36256 | 0.10925 | 0.37107 | 0.75029 | | | | 0.33124 | |
| ***k* = 4** | n | Within Cluster Sum of Square | Average Distance | Maximum Distance | Distance Between Clusters | | | | | |
|  |  |  |  |  | Cluster 1 | | Cluster 2 | | | Cluster 3 |
| Cluster1 | 11 | 0.04722 | 0.05894 | 0.13184 | - | | 0.68446 | | | 0.3532 |
| Cluster2 | 20 | 0.36256 | 0.10925 | 0.37107 | 0.68446 | | - | | | 0.33124 |
| Cluster3 | 36 | 0.25237 | 0.06903 | 0.1695 | 0.35322 | | 0.33124 | | | - |
| Cluster4 | 2 | 0.0239 | 0.10931 | 0.10931 | 0.42789 | | 1.11236 | | | 0.78112 |
| ***k* = 5** | n | Within Cluster Sum of Square | Average Distance | Maximum Distance | Distance Between Clusters | | | | | |
|  |  |  |  |  | Cluster 1 | Cluster 2 | | Cluster 3 | | Cluster 4 |
| Cluster1 | 17 | 0.07558 | 0.05703 | 0.11522 | - | 0.24423 | | 0.27818 | | 1.01354 |
| Cluster2 | 33 | 0.19657 | 0.06545 | 0.15768 | 0.24423 | - | | 0.52241 | | 0.7693 |
| Cluster3 | 6 | 0.05002 | 0.06604 | 0.19171 | 0.27818 | 0.52241 | | - | | 1.29171 |
| Cluster4 | 2 | 0.0239 | 0.10931 | 0.10931 | 1.01354 | 0.7693 | | 1.29171 | | - |
| Cluster5 | 11 | 0.04722 | 0.05894 | 0.13184 | 0.58564 | 0.34141 | | 0.86382 | | 0.42789 |

Average distance is equal to the average Euclidean distance of individual data points to the cluster center within a cluster. Distance between clusters is equal to the Euclidean distance between cluster centers.
